# Drug-Coated Balloon Treatment for Delayed Recanalization of Symptomatic Intracranial Artery Occlusion

**DOI:** 10.1007/s12975-022-01024-5

**Published:** 2022-04-23

**Authors:** Wei Zhao, Xi Chu, Yun Song, Jinping Zhang, Lili Sun, Meimei Zheng, Hao Yin, Jun Zhang, Wei Wang, Yao Meng, Ju Han

**Affiliations:** Department of Neurology, The First Affiliated Hospital of Shandong First Medical University & Shandong Provincial Qianfoshan Hospital, No. 16766#, Jingshi Road, Jinan, China

**Keywords:** Non-acute intracranial artery occlusion, Drug-coated balloon, Delayed recanalization, Outcome

## Abstract

Patients with medically refractory non-acute intracranial artery occlusion (ICAO) are difficult to treat. The optimal intervention for these patients is not known. We evaluated the feasibility and safety of drug-coated balloon (DCB) treatment for non-acute ICAO. Consecutive patients with symptomatic medically refractory atherosclerotic non-acute ICAO from January 2015 to July 2021 who underwent DCB treatment were retrospectively analyzed. The rates of stroke, transient ischemic attack, and death within 30 days and the follow-up results were evaluated. A total of 148 patients were enrolled in this study. The 30-day rate of stroke, transient ischemic attack, and death was 8.8%. During the 25.8 ± 15.8-month clinical follow-up period, the rate of outcome beyond 30 days was 4.7%. In the 66 patients with vessel imaging follow-up, 13.6% (9/66) had restenosis. The present study suggests that DCB dilatation is a feasible and effective alternative in carefully selected patients with symptomatic non-acute ICAO.

## Introduction

Intracranial artery occlusion (ICAO) is a very important cause of ischemic stroke that carries a high risk of recurrent symptoms, high morbidity, and high mortality despite aggressive clinical management [1–4]. Recanalization treatment for acute ischemic stroke attributed to ICAO, including intravenous and endovascular approaches, is provided within specific time windows. However, a large number of these patients are unable to reach a qualified stroke center within the specified times after symptom onset. A proportion of patients with ICAO endure the acute arterial occlusion, which transforms into the non-acute stage. A considerable number of these patients, especially patients with hemodynamic damage, present with progressive or recurrent ischemic stroke despite optimal medical therapy. Non-acute ICAO is especially challenging to treat with interventions, and the optimal intervention has not been determined.

Drug-coated balloons (DCBs) were developed to address the concern of restenosis that is associated with traditional interventions. DCBs are coated with the antiproliferative agent paclitaxel, and dilation of the DCB induces drug release from the balloon surface into the vessel wall to inhibit intimal hyperplasia [5–8].

DCBs reduce the risk of restenosis, long-term dual antiplatelet therapy (DAPT), and the need for an additional stent, which decrease the risk of recurrent stroke and hemorrhage events and benefit patients who require other surgical treatments [9–11]. There are few clinical reports on the safety and effectiveness of DCBs for the treatment of non-acute ICAO [12].The present study evaluated the safety and efficacy of paclitaxel DCBs in the treatment of real-world subjects with symptomatic non-acute ICAO.

## Materials and Methods

### Study Population

We retrospectively reviewed our prospective stroke database to identify consecutive patients with symptomatic non-acute ICAO who were treated with DCBs from January 2015 to July 2021. Informed consent was obtained from all patients or their authorized family members before surgery, and the study was approved by our institutional review board and complied with the Declaration of Helsinki.

ICAO was diagnosed using CT angiography (CTA) or magnetic resonance angiography (MRA) and confirmed by digital subtraction angiography (DSA) for all patients. The duration of occlusion was defined as the time from initial radiological diagnosis to endovascular treatment. The following inclusion criteria were used: (1) duration of occlusion > 24 h; (2) intracranial atherosclerosis was the primary etiology; (3) recurrent transient ischemic attacks (TIA) or stroke related to an occluded intracranial artery despite optimal medical treatment, which was defined as treatment that included DAPT, statin, blood pressure and glucose control, smoking cessation, and an emphasis on a healthy lifestyle; and (4) hemodynamic failure and hypoperfusion in the target artery territory were confirmed based on clinical and imaging evidence (the symptoms were triggered or exacerbated by orthostatism or lower blood pressure, and relatively small and multiple infarctions on diffusion-weighted imaging (DWI) with a large area of low perfusion assessed using arterial spin labeling (ASL), which demonstrated hypoperfusion defects in the target territory of these patients). The following exclusion criteria were used: (1) nonatherosclerotic diseases, such as suspected cerebral vasculitis, arterial dissection, and potential source of cardiac embolism; (2) clinical symptoms were stable with optimal medical treatment; (3) concomitant with intracranial aneurysms and any bleeding disorder; (4) life expectancy < 1 year due to other medical conditions; and (5) contraindications to surgery, such as known allergy or contraindication to aspirin, clopidogrel, or anesthesia.

### Procedure

**Fig. 1:**
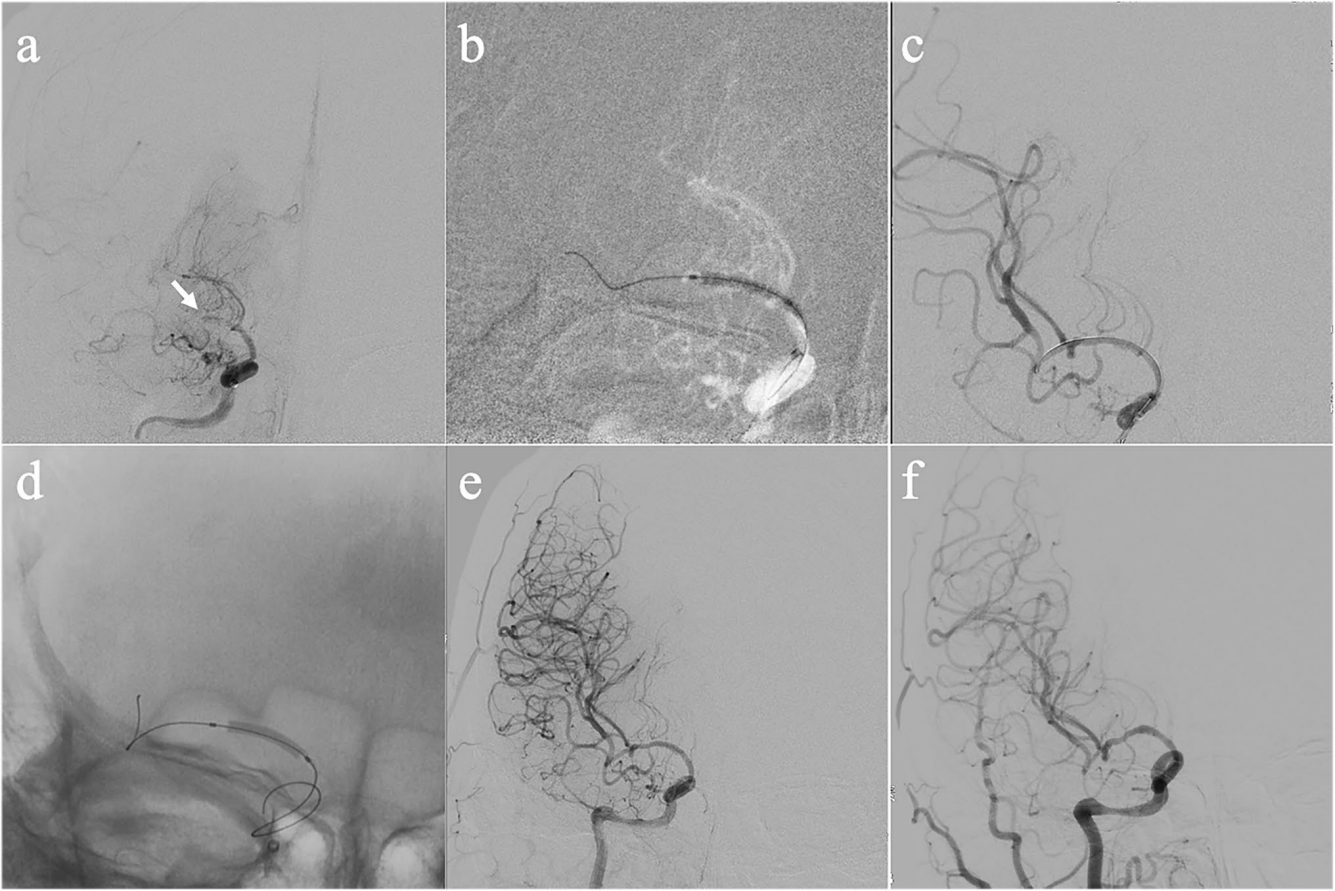
Angiographic outcome of DCB dilation for ICAO. Angiographic outcomes of DCB dilation for a patient with occlusion of middle cerebral artery and follow-up. **a** Middle cerebral artery occlusion (the arrow indicates the occlusion site). **b** Predilation with a conventional balloon. **c** Angiographic result after predilation. **d** DCB dilation. **e** Angiographic outcome after DCB dilation. **f** Angiographic outcome at 6-month follow-up

**Fig. 2:**
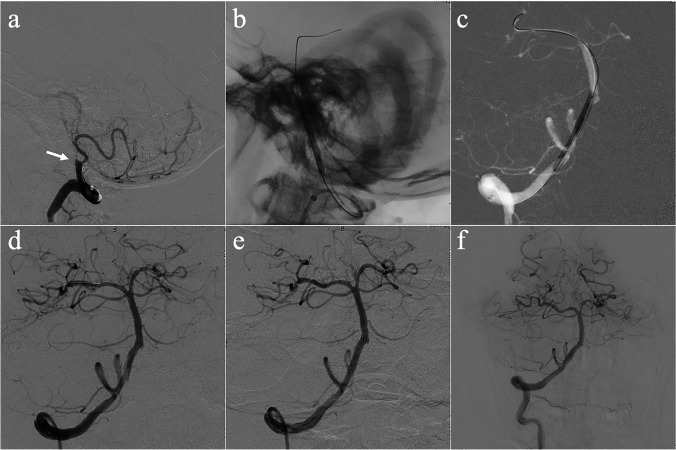
Angiographic outcome of DCB dilation and remedial stenting for ICAO. Angiographic outcomes of DCB dilation and remedial stenting for a patient with occlusion of intracranial vertebral artery and follow-up. **a** Intracranial vertebral artery occlusion (the arrow indicates the occlusion site). **b** Predilation with a conventional balloon. **c** DCB dilation. **d** Angiographic result after DCB dilation with vessel dissection. **e** Angiographic outcome after remedial stenting implantation. **f** Angiographic outcome at 6-month follow-up

DAPT with 100 mg aspirin and 75 mg clopidogrel daily was given at least 5 days before the procedure. Thromboelastography platelet mapping was performed to guide the modulation of antiplatelet treatment. The endovascular procedures were performed under general anesthesia. The details of the procedure were described previously [12–14]. Adequate predilation using conventional balloons (Gateway; Boston Scientific, USA) was mandatory before DCB. DCB (SeQuent Please; B. Braun, Germany) dilation was only performed when the residual stenosis was not greater than 50% or there was no dissection after predilation. The diameter of the DCB corresponded to approximately 80 to 100% of the diameter of the normal vessel and was 0.5 to 1 mm larger than the conventional balloon. The DCB covered the entire lesion and was slowly inflated at a nominal pressure for 60 s to transit the paclitaxel into the vessel wall. After withdrawal of the DCB, an angiogram was reperformed to evaluate the lumen and exclude vessel dissection, perforation, and distal embolization (Fig. 1). If the residual stenosis was > 50% or there was vessel dissection after DCB dilation, remedial stenting implantation was performed (Fig. 2). The choice of stent (self-expanding or balloon-mounted stent) and the use of glycoprotein IIb/IIIa inhibitors were left to the surgeon’s discretion. Post-procedural antegrade flow was graded using the TICI grading system, and technical success was determined by recanalization with a TICI grade 2b on post-procedural angiography. DAPT was maintained for 3 months for patients with only DCB dilation and 6 months for patients with remedial stenting implantation. Aspirin or clopidogrel monotherapy was maintained thereafter.

### Data Collection and Follow-up Outcomes

Demographic, clinical, angiographic, and periprocedural data were collected. The baseline modified Rankin Scale (mRS) and the National Institutes of Health Stroke Scale (NIHSS) were used before the procedure. The Alberta Stroke Program Early CT score (ASPECTS) for anterior circulation and posterior circulation acute stroke prognosis early CT score (pc-ASPECTS) for posterior circulation on the last DWI before the procedure were assessed by well-trained neurologists. The patients were scheduled to return for an angiographic examination 6 months (± 1 month) after the index procedure. The primary outcome within 30 days was any stroke (including ischemic or hemorrhagic stroke), TIA, and death after the procedure. The primary outcomes beyond 30 days were ischemic stroke within the territory of the target vessel, TIA within the territory of the target vessel, hemorrhagic stroke, rate of restenosis, and death attributed to stroke during the follow-up post-index procedure.

Restenosis was defined as > 50% stenosis within or immediately adjacent (within 5 mm) to the treated segment and > 20% absolute luminal loss. Symptomatic restenosis was defined as restenosis associated with ischemic symptoms of the offending vessel territory. Two investigators reviewed imaging and clinical outcomes. Disagreements were resolved via consensus.

### Statistical Analysis

Continuous data are expressed as the means ± SD or as the medians with interquartile range (IQR), and categorical data are presented as numbers and percentages. All statistical analyses were performed using SPSS version 22.0 for Windows (IBM, Armonk, New York, USA).

## Results

A total of 148 patients treated with DCBs for delayed recanalization of symptomatic intracranial artery occlusion were enrolled between January 2015 and July 2021. The baseline and clinical characteristics of the patients are shown in Table 1. One hundred (67.6%) patients were men, and the average age was 58.0 ± 9.1 years. The most common risk factor was hypertension in 80.4% of all the patients. The median baseline NIHSS score was 2 (IQR, 1–6.25), and the median baseline mRS score was 2 (IQR, 1–3.25). The median ASPECTS of the 108 anterior circulation patients was 8 (IQR, 6–9), and the median PC-ASPECTS of the 40 posterior circulation patients was 8 (IQR, 6–9) before the procedure. Procedural success was achieved in 100.0% (*n* = 148) of lesions. The median time from symptom onset to treatment was 29 days, and the median time from image documentation of occlusion to treatment was 15 days. Remedial stents were implanted in 35.1% (*n* = 52 of 148) of lesions.

**Table 1:** Baseline and clinical characteristics of the patients

Characteristic	*n* = 148 (%)
Age, y, mean (SD)	58.0 ± 9.1
Male	100 (67.6)
Hypertension	119 (80.4)
Diabetes mellitus	59 (40.0)
Hyperlipidemia	26 (17.6)
Coronary artery disease	32 (21.6)
Atrial fibrillation	5 (3.4)
Ischemic stroke history	38 (25.7)
Smoking	69 (46.6)
Qualifying ischemic events	
TIA	3 (2.0)
Stroke	145 (98.0)
Symptomatic qualifying artery	
Intracranial carotid artery	26 (17.6)
Middle cerebral artery	82 (55.4)
Basilar artery	18 (12.2)
Intracranial vertebral artery	22 (14.9)
Symptom onset to treatment (days), median (IQR)	29 (19–60)
Image-documented occlusion to treatment (days), median (IQR)	15 (7–28)
Successful revascularization, %	148 (100)
Remedial stenting	52 (35.1)
Stenosis degree after intervention, %, median (IQR)	0 (0–20.0)

*TIA*, transient ischemic attacks; *IQR*, interquartile range

### The 30-Day Outcomes

The 30-day outcome rate was 8.8% (13/148; Table 2). Ischemic stroke occurred in 5 patients (3.4%), and all strokes were related to the target territory. Three patients presented with perforator ischemic stroke related to the basilar artery territory, 1 patient presented with perforator ischemic stroke related to the intracranial vertebral artery territory, and embolization occurred in 1 patient related to the intracranial carotid territory. Hemorrhagic stroke occurred in 5 patients (3.4%), and all cases were related to the target middle cerebral artery territory, which resulted from hyperperfusion after recanalization. Death occurred in 3 patients (2.0%), and all 3 deaths were attributed to hyperperfusion after target lesion recanalization (2 lesions in the middle cerebral artery and 1 lesion in the basilar artery).

**Table 2:** Thirty-day safety outcomes and follow-up outcomes for the patients

Characteristic	*n* = 148 (%)
Thirty-day safety outcomes	
Stroke, TIA, and death, %	13 (8.8)
Ischemic stroke, %	5 (3.4)
Hemorrhagic stroke, %	5 (3.4)
TIA, %	0 (0.0)
Death, %	3 (2.0)
Follow-up time (months), mean (SD)	25.8 ± 15.8
Stroke, TIA, and death, %	7 (4.7)
Ischemic stroke, %	4 (2.7)
Hemorrhagic stroke, %	1 (0.7)
TIA, %	2 (1.4)
Death attributed to stroke, %	0 (0)
Angiographic outcomes	
Imaging follow-up, %	66 (44.6)
Imaging follow-up time (months), mean (SD)	4.9 ± 2.4
Restenosis on follow-up image, %	9 (13.6)
Symptomatic restenosis	6 (9.0)

*TIA*, transient ischemic attacks; *IQR*, interquartile range

### Follow-up Outcomes Beyond 30 Days

Clinical and angiographic follow-up outcomes of the patients beyond 30 days are presented in Table 2. During the 25.8 ± 15.8-month clinical follow-up period, the rate of outcome beyond 30 days was 4.7% (7/148).

Ischemic stroke occurred in 4 patients (2.7%), and TIA occurred in 2 patients (1.4%). All 6 of these patients had imaging follow-up, and they all had target lesion restenosis. Hemorrhagic stroke occurred in 1 patient (0.7%). There was no death attributed to stroke during the follow-up period. During the 4.9 ± 2.4-month vessel imaging follow-up period, DSA was obtained for 55 patients, MRA was obtained for 7 patients, and CTA was obtained for 4 patients. Restenosis occurred in 9 patients (9/66, 13.6%) who had follow-up imaging: 6 patients presented with angiographic symptomatic restenosis, and the other 3 patients presented with asymptomatic restenosis.

## Discussion

Non-acute ICAOs are among the most challenging types of lesions to treat using endovascular strategies. Previous clinical and animal studies suggest a long-lasting existence of penumbra after non-recanalized stroke, and delayed reperfusion after ischemic stroke may improve neurological function by recovering blood flow into the penumbra [15–23]. Clinical data on the safety and effectiveness of endovascular therapies for the treatment of non-acute ICAOs are limited. The present study is the largest clinical analysis to evaluate DCBs for the treatment of non-acute ICAOs, and we preliminarily demonstrated the feasibility and efficacy of DCB dilatation for symptomatic non-acute ICAOs.

Procedural success was achieved in all patients. The primary outcome rate within 30 days was 8.8%. Ischemic stroke occurred in 3.4% of patients, and most cases were perforator strokes related to the posterior circulation. Hemorrhagic stroke occurred in 3.4% of patients and resulted from hyperperfusion after middle cerebral artery recanalization. Three deaths (2.0%) occurred because of hyperperfusion after target lesion recanalization (2 middle cerebral arteries, and 1 basilar artery). Our study showed that endovascular recanalization for posterior circulation occlusion was associated with a higher risk of perforator stroke, and the hyperperfusion risk was higher for anterior circulation occlusion recanalization, which is consistent with previous studies on angioplasty and stenting for intracranial stenosis.

The event rate (4.7%) remained low over the average 25.8-month clinical follow-up period beyond the 30-day post-index procedure in these patients. Ischemic stroke and TIA occurred in 4.1% of patients and were related to the target lesion restenosis. Hemorrhagic stroke occurred in 1 patient (0.7%).

The in-stent restenosis rate may be as high as 32% for bare metal stents, which accounts for 39% of recurrent ischemic symptoms [24]. Therefore, promising treatments with a minimum risk of restenosis options for ICAOs must be examined. During the average 4.9-month vessel imaging follow-up period, DSA was obtained for 55 patients, MRA was obtained for 7 patients, and CTA was obtained for 4 patients. Restenosis occurred in 13.6% of patients who had follow-up imaging: 6 patients had symptomatic restenosis, and the other 3 patients had asymptomatic restenosis. Although we required all of the patients to undergo DSA 6 months after the procedure, imaging follow-up was not compulsory. Many patients were reluctant to undergo DSA because they were without symptoms, and there may be patient selection bias. Therefore, the real rate of restenosis may be lower than 13.6%. The fact that the restenosis rate in our study was much lower than previous studies for bare metal stents indicates the promising potential of DCB treatment for ICAO. However, the rate of angiographic follow-up was too low to make a significant conclusion.

Our data supported the fact that most adverse events occurred within the first few weeks after the procedure in patients who underwent DCB treatment. Considering the poor natural history of ICAO with hemodynamic damage (annual ipsilateral stroke incidence of 23.7%) [25], recanalization is worth attempting despite the risk of failure and complications in patients treated with DCBs because it is an alternative option for medically refractory non-acute ICAO. Further studies are needed to examine which patients are the best candidates and perform individualized risk stratification.

### Limitations

This study has some limitations. First, this study was a retrospective cohort study. Second, this study was a single-center study, and the choice of treatment method was based on patient preference and surgeon experience, which may lead to selection bias. Third, this study did not have a control group. We will compare DCB treatment with conventional balloon dilatation/stenting or no endovascular treatment in the future.

## Conclusion

Paclitaxel DCB treatment was feasible and effective for symptomatic non-acute ICAOs. Further studies are needed to explore individualized risk stratification and select low-risk candidates for treatment. Prospective studies are needed to investigate whether DCB treatment compares favorably with aggressive medical management in these patients.

## Data Availability

All the data that support the findings of this study are available from the corresponding author on reasonable request.
